# Different radiomics annotation methods comparison in rectal cancer characterisation and prognosis prediction: a two-centre study

**DOI:** 10.1186/s13244-024-01795-5

**Published:** 2024-08-26

**Authors:** Ying Zhu, Yaru Wei, Zhongwei Chen, Xiang Li, Shiwei Zhang, Caiyun Wen, Guoquan Cao, Jiejie Zhou, Meihao Wang

**Affiliations:** Department of Radiology, The First Affiliated Hospital of Wenzhou Medical University, Wenzhou, China

**Keywords:** Rectal cancer, MRI, Radiomics, Annotation methods

## Abstract

**Objectives:**

To explore the performance differences of multiple annotations in radiomics analysis and provide a reference for tumour annotation in large-scale medical image analysis.

**Methods:**

A total of 342 patients from two centres who underwent radical resection for rectal cancer were retrospectively studied and divided into training, internal validation, and external validation cohorts. Three predictive tasks of tumour T-stage (pT), lymph node metastasis (pLNM), and disease-free survival (pDFS) were performed. Twelve radiomics models were constructed using Lasso-Logistic or Lasso-Cox to evaluate and four annotation methods, 2D detailed annotation along tumour boundaries (2D), 3D detailed annotation along tumour boundaries (3D), 2D bounding box (2D_BB_), and 3D bounding box (3D_BB_) on T2-weighted images, were compared. Radiomics models were used to establish combined models incorporating clinical risk factors. The DeLong test was performed to compare the performance of models using the receiver operating characteristic curves.

**Results:**

For radiomics models, the area under the curve values ranged from 0.627 (0.518–0.728) to 0.811 (0.705–0.917) in the internal validation cohort and from 0.619 (0.469–0.754) to 0.824 (0.689–0.918) in the external validation cohort. Most radiomics models based on four annotations did not differ significantly, except between the 3D and 3D_BB_ models for pLNM (*p* = 0.0188) in the internal validation cohort. For combined models, only the 2D model significantly differed from the 2D_BB_ (*p* = 0.0372) and 3D models (*p* = 0.0380) for pDFS.

**Conclusion:**

Radiomics and combined models constructed with 2D and bounding box annotations showed comparable performances to those with 3D and detailed annotations along tumour boundaries in rectal cancer characterisation and prognosis prediction.

**Critical relevance statement:**

For quantitative analysis of radiological images, the selection of 2D maximum tumour area or bounding box annotation is as representative and easy to operate as 3D whole tumour or detailed annotations along tumour boundaries.

**Key Points:**

- There is currently a lack of discussion on whether different annotation efforts in radiomics are predictively representative.
- No significant differences were observed in radiomics and combined models regardless of the annotations (2D, 3D, detailed, or bounding box).
- Prioritise selecting the more time and effort-saving 2D maximum area bounding box annotation.

**Graphical Abstract:**

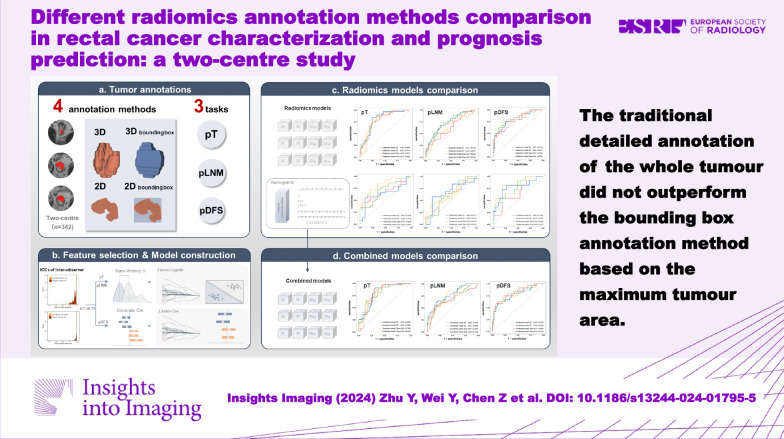

## Introduction

Colorectal cancer ranks as the third most common cancer worldwide and it is the second leading cause of cancer-related deaths [1]. Rectal cancer represents approximately one-third of these cases. Rectal cancer management is complex, and accurate prediction of tumour T-stage and the status of lymph node metastasis (LNM) are essential for treatment decision-making and prognosis [2]. T2-weighted images (T2WI), as one of the most important sequences of rectal magnetic resonance imaging (MRI), have excellent soft-tissue contrast. When used in image quantitative analysis, like radiomics, T2WI plays an important role in rectal cancer preoperative staging diagnosis, evaluation of the efficacy of neoadjuvant therapy, and prognosis prediction [3–5].

Radiomics, an emerging image quantitative analysis technology, can extract numerous quantitative features from medical images, allowing non-invasive profiling of tumour heterogeneity by correlating multiple features with potential biological outcomes [6, 7]. Studies of tumour heterogeneity based on radiomics, such as lesion diagnosis and risk stratification, have shown promising predictive values in oncology practice [8–11].

Radiomics workflow involves image acquisition, tumour annotation, feature extraction, feature selection, and model construction [4, 12]. Tumour annotation is important in ensuring the repeatability, prediction accuracy, and research efficiency of radiomics quantitative analysis [13]. However, there are two main controversies in the tumour annotation stage. First, tumours often cover multiple layers in MRI. Yang et al [14], suggested that the whole tumour slices (3D) annotation of non-small cell lung cancer might better predict survival rates than the maximum tumour area (2D), whereas others have found that there is no significant difference in prediction between non-small cell lung cancer and gastric cancer using 2D or 3D annotations [15, 16]. It has also been suggested that manual annotation of tumour boundaries is subjective and time-consuming, while rough bounding box annotation with good efficiency and reproducibility shows the ability of annotation and obtains valuable information about the peritumoural microenvironment [17, 18]. 3D annotation may provide more comprehensive tumour information in some cases and contribute to a more accurate prognosis but is not superior to 2D annotation in all cases. Meanwhile, bounding box annotation, as a fast and reproducible method, provides sufficient information, although it may sacrifice tumour shape information and may be more efficient in practical applications [17]. It is always a dilemma whether to use 3D or 2D annotation and whether to annotate along the tumour boundaries or within the bounding box in radiomics-based oncology practice [13].

Therefore, the objective of this two-centre study is to compare the performance of four annotation methods in radiomics modelling predicting T-stage (pT), LNM (pLNM), and disease-free survival (pDFS) in rectal cancer to help determine the best practices for radiomics tumour annotation. These four annotation methods based on T2WI include 2D, 3D, maximum tumour area bounding box annotation (2D_BB_), and whole tumour bounding box annotation (3D_BB_).

## Materials and methods

### Patients

**Fig. 1:**
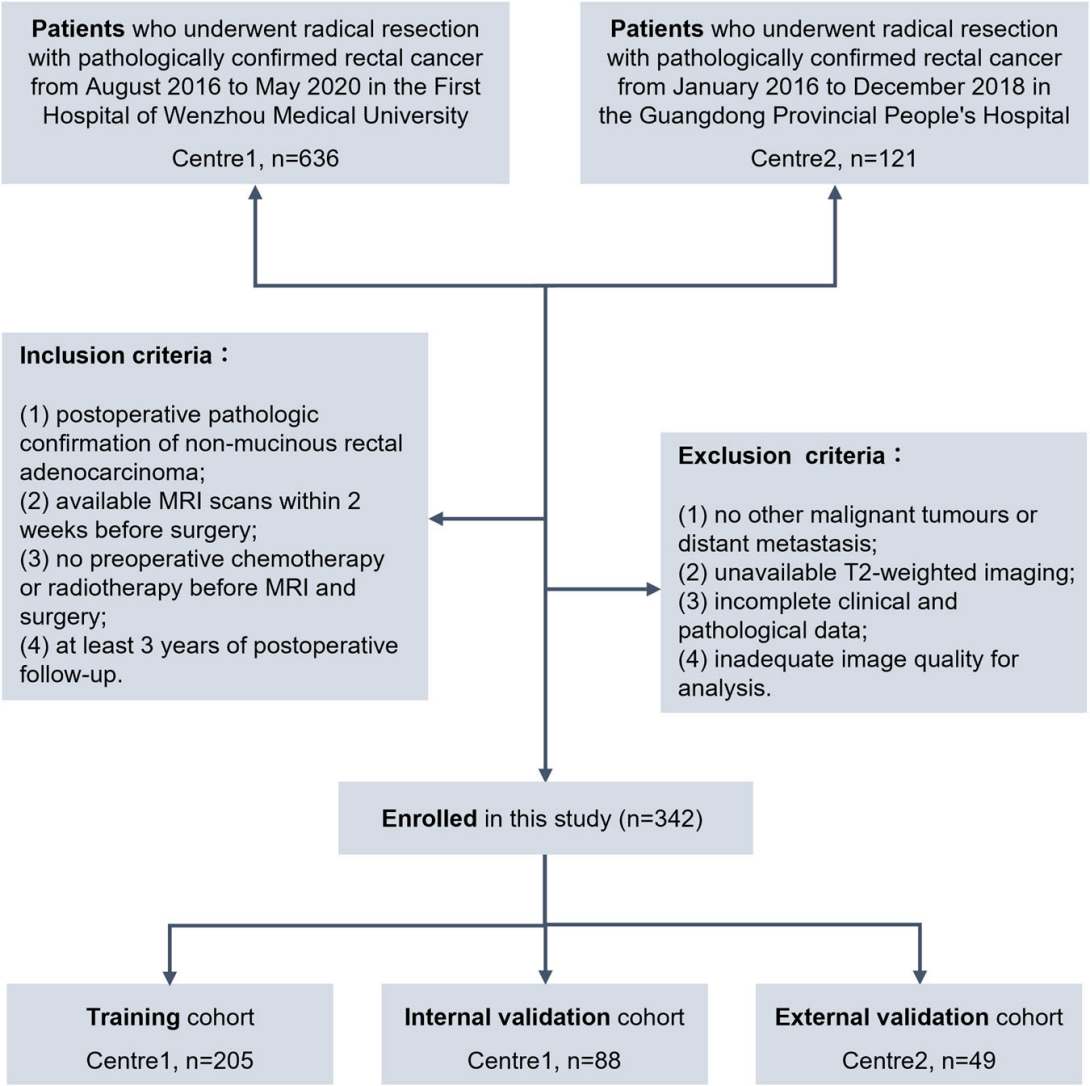
Patient flowchart of this study

Seven hundred and fifty-seven patients with pathologically confirmed rectal cancer were retrospectively reviewed from two centres, including 636 from Centre1 and 121 from Centre2. The inclusion criteria included: (1) postoperative pathologic confirmation of non-mucinous adenocarcinoma; (2) available MRI scans within 2 weeks before surgery; (3) no preoperative chemotherapy or radiotherapy before MRI and surgery; and (4) at least 3 years of postoperative follow-up. The exclusion criteria included: (1) no other malignant tumours or distant metastasis; (2) unavailable high-resolution T2-weighted imaging; (3) incomplete clinical and pathological data; and (4) inadequate image quality for analysis. Finally, a total of 342 patients were enrolled in the study. Two hundred and ninety-three patients from the First Hospital of Wenzhou Medical University (Centre1) were randomised 7:3 into the training cohort (*n* = 205) and internal validation cohort (*n* = 88). Forty-nine patients from the Guangdong Provincial People’s Hospital (Centre2) were held out for the external validation cohort. Figure 1 shows the patient selection process and the case numbers for training and internal and external validation cohorts. The collection of clinicopathological and prognostic parameters is shown in Supplementary Method S1.

### MRI acquisitions and parameters

Achieva 3.0-T scanners (Philips Medical, the Netherlands) were used in Centre1, and Ingenia 3.0-T scanners (Philips Medical, the Netherlands) were used in Centre2. For Centre1, axial T2WI without fat saturation was performed using the following protocol: TR/TE 3000–5000/80; Field of View (FOV) 230 × 152 mm^2^; Matrix size 460 × 302; section thickness 3 mm. For Centre2, the parameters were: TR/TE 4300/141.12; FOV 100 mm^2^; Matrix size 464 × 461; section thickness 4 mm. The MRI protocol details have been previously documented in Supplementary Table S.1.

### Tumour annotations and radiomics feature extraction

**Fig. 2:**
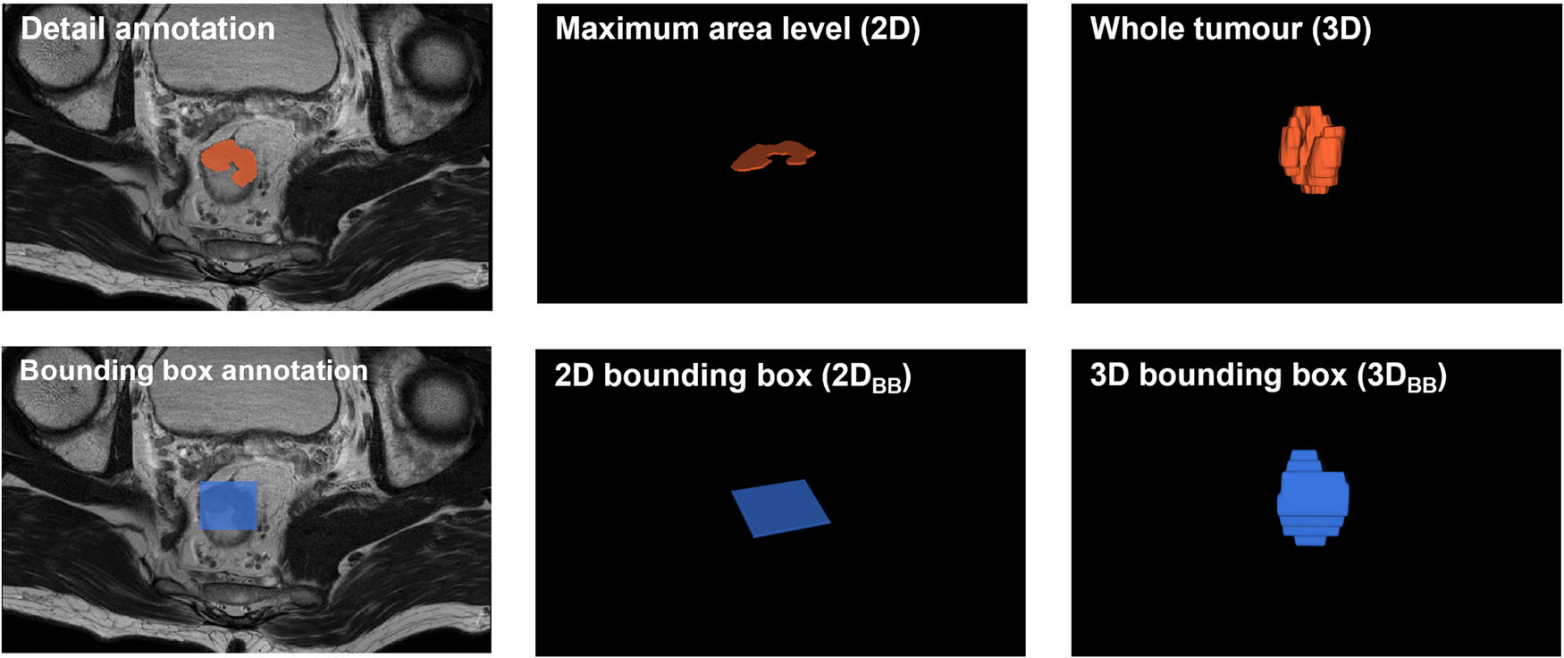
Example of four annotations in a 52-year-old patient with rectal cancer

A junior radiologist (Y.Z.) reviewed all T2WI slices and annotated 3D regions of interest (ROI) of the whole tumour using ITKSNAP software (https://www.itksnap.org). The slice with the maximum tumour area was identified by an automated algorithm, defining the 2D ROI. Both 3D and 2D ROIs were expanded to form minimal BB, labelled as 3D_BB_ and 2D_BB_ ROIs, respectively, as shown in Fig. 2.

The Pyradiomics 3.0.1 python package was used to extract four radiomics feature sets from the ROIs, derived through four annotations [19]. The feature extraction configuration included resampling (1 × 1 × 1) with a bin width of 25. The same features were extracted from all ROIs, including the original, Laplacian of Gaussian (LoG) and wavelet filter images. Each image category contains six classes. All features comply with the Imaging Biomarker Standardization Initiative criteria [20]. To standardise feature values, z-score normalisation was applied to reduce the impact of different grey level ranges. The SMOTE technique was employed to achieve data balance.

### Feature selection and twelve radiomics modelling

The selection of the optimal radiomics features involved a three-step process. Initially, the interclass correlation coefficient (ICC) was calculated to evaluate feature reproducibility. Specifically, an intermediate radiologist (Z.C.) randomly selected and re-annotated the whole tumour ROIs on T2WI, and the largest slice was automatically identified to generate the maximum tumour area ROI. The re-annotated whole tumour ROIs and automatically generated maximum tumour area ROI were subjected to ICC test to assess stability concerning whole tumour (3D and 3D_BB_) and maximum tumour area (2D and 2D_BB_) features. Features with the ICC > 0.75 were included for subsequent analysis.

Next, for the pT and pLNM tasks, the Mann–Whitney *U* test was used to identify features with *p* < 0.1. To maintain a balance in the number of features at this stage, univariate Cox regression was utilised in the pDFS task, and features with *p* < 0.05 were selected for further analysis.

Finally, we used the “glmnet” package in R to fit Lasso-Logistic and Lasso-Cox regression, identifying the best predictive features among the remaining features (Supplementary Method S2). To minimise estimation bias, we used 10-fold cross-validation to determine the optimal value of the penalty function λ and compressed the coefficients of irrelevant features to obtain the optimal subset of features with non-zero correlation coefficients [21]. The process of determining the λ-value and coefficients using Lasso is shown in Supplementary Fig. S.1. The best features retained were linearly combined, using the correlation coefficients as weighting factors to produce the corresponding models. The Radiomic scores (Rad-scores) for each patient were calculated, including predicting T-stage by 2D detail annotation (2D^pT^), predicting T-stage by 3D detail annotation (3D^pT^), predicting T-stage by 2D_BB_ annotation (${2\text{D}}_{\text{BB}}^{\text{pT}}$), predicting T-stage by 3D_BB_ annotation (${3\text{D}}_{\text{BB}}^{\text{pT}}$); predicting LNM by 2D detail annotation (2D^pLNM^), predicting LNM by 3D detail annotation (3D^pLNM^), predicting LNM by 2D_BB_ annotation (${2\text{D}}_{\text{BB}}^{\text{pLNM}}$), predicting LNM by 3D_BB_ annotation (${3\text{D}}_{\text{BB}}^{\text{pLNM}}$); predicting DFS by 2D detail annotation (2D^pDFS^), predicting DFS by 3D detail annotation (3D^pDFS^), predicting DFS by 2D_BB_ annotation (${2\text{D}}_{\text{BB}}^{\text{pDFS}}$), predicting DFS by 3D_BB_ annotation (${3\text{D}}_{\text{BB}}^{\text{pDFS}}$).

Feature contributions were assessed using SPSS multivariate Logistic and Cox regression by Wald test. The three features with the lowest *p*-values were identified as the most influential features.

### Construction, evaluation, and validation of combined models

For different prediction tasks, we first applied the Mann–Whitney *U* test or univariate Cox regression test, to identify statistically significant clinical factors and calculate the odds or hazard ratios (ORs/HRs) [22]. Subsequently, the Rad-scores generated by four annotations were combined with the selected clinical factors for each task to construct combined models in the form of nomograms. Combined models were then validated by the internal validation cohort. The clinical relevance of the models was assessed by decision curve analysis (DCA).

### Statistical analysis

For continuous variables, we used either a *t*-test or the Mann–Whitney *U* test, and for categorical variables, we used either the chi-square test or Fisher’s exact test. Statistical analyses were performed with SPSS (version 27.0, IBM, Armonk, NY, USA), MedCalc (version 19.8, Mariakerke, Belgium), and R package (version 4.1.3, Vienna, Austria). We used receiver operating characteristic (ROC) curves and computed the area under the curve (AUC) to evaluate the predictive performance. The DeLong test was used to compare the performance of the two annotation models for each task. Bilateral *p* < 0.05 was considered statistically difference significant unless otherwise specified.

In the pDFS survival analysis, statistical correlations between clinical factors were determined by univariate and multivariate Cox regression. The significant clinical risk factors were then integrated with Rad-scores into nomograms to create combined models. Harrell’s concordance index (C-index) values and 3-year ROC were calculated. The patients’ Rad-scores will be divided into low-risk and high-risk groups using the Kaplan–Meier [23] with the median as the cutoff point. Finally, the Log-rank test was used to ascertain significant differences between the two risk groups in the pDFS combined models.

## Results

### Patient characteristics

A total of 342 patients were enrolled in the study from two centres, including the training (*n* = 205), internal validation (*n* = 88), and external validation cohort (*n* = 49). Demographic and clinical characteristics of patients are listed in Table 1. Age, circumferential resection margin, extramural venous invasion, tumour length, and LNM assessed by MRI were significantly different in the three cohorts from the two centres.

**Table 1:** Clinical characteristics of patients

Characteristics	Training cohort (*n* = 205)	Internal validation cohort (*n* = 88)	External validation cohort (*n* = 49)	*p*-value
Age (years), mean ± SD	65.2 ± 10.7	65.9 ± 11.2	59.1 ± 12.5	**0.001**
Sex, No.(%)				0.110
Male	137 (66.8%)	54 (61.4%)	25 (51.0%)	
Female	68 (33.2%)	34 (38.6%)	24 (49.0%)	
BMI, mean ± SD	23.02 ± 2.91	23.24 ± 2.40		0.525
CEA level, No. (%)				0.898
< 5	69 (33.7%)	29 (33.0%)	18 (36.7%)	
≥ 5	136 (66.3%)	59 (77.0%)	31 (63.3%)	
PR, No. (%)				0.942
Upper	21 (10.2%)	10 (11.4%)		
Middle	85 (41.5%)	35 (39.8%)		
Low	99 (48.3%)	43 (48.9%)		
CRM, No. (%)				**0.002**
Absent	183 (89.3%)	80 (90.9%)	35 (71.4%)	
Present	22 (10.7%)	8 (9.1%)	14 (28.6%)	
EMVI, No. (%)				**0.002**
Negative	177 (86.3%)	80 (90.9%)	34 (69.4%)	
Positive	28 (13.7%)	8 (9.1%)	15 (30.6%)	
Tumour length (mm), mean ± SD	37.95 ± 15.33	39.03 ± 16.86	40.90 ± 14.24	**0.020**
MR-T stage, No. (%)				0.794
T1-2	85 (41.5%)	40 (45.5%)	20 (40.8%)	
T3-4	120 (58.5%)	48 (54.5%)	29 (59.2%)	
MR-LNM, No. (%)				**< 0.001**
Yes	151 (73.7%)	62 (70.5%)	20 (40.8%)	
No	54 (26.3%)	26 (29.5%)	29 (59.2%)	
p-T stage*, No. (%)				0.244
T1-2	101 (49.3%)	49 (55.7%)	20 (40.8%)	
T3-4	104 (50.7%)	39 (44.3%)	29 (59.2%)	
p-LNM*, No. (%)				0.216
Yes	122 (59.5%)	54 (61.4%)	23 (46.9%)	
No	83 (40.5%)	34 (38.6%)	26 (53.1%)	
TDs, No. (%)				0.641
Negative	178 (86.8%)	75 (85.2%)	40 (81.6%)	
Positive	27 (13.2%)	13 (14.8%)	9 (18.4%)	
LVI, No. (%)				0.554
Negative	156 (76.1%)	71 (80.7%)	40 (81.6%)	
Positive	49 (23.9%)	17 (19.3%)	9 (18.4%)	
PI, No. (%)				0.935
Negative	152 (74.1%)	64 (72.7%)	37 (75.5%)	
Positive	53 (25.9%)	24 (27.3%)	12 (24.5%)	
Histologic grade, No. (%)				**< 0.001**
High	18 (8.8%)	5 (5.7%)		
Middle	42 (20.5%)	16 (18.2%)		
High-Middle	141 (68.8%)	66 (75.0%)	45 (91.8%)	
Low	4 (2.0%)	1 (1.1%)	4 (8.2%)	
Local and distant metastasis, No. (%)				0.785
Yes	60 (29.3%)	24 (27.3%)	12 (24.5%)	
No	145 (70.7%)	64 (72.7%)	37 (75.5%)	
DFS* (years), median (IQR)	3.38 (2.40, 3.98)	3.49 (2.61, 4.24)	3.58 (3.01, 4.23)	0.060

Boldface indicates statistical significance (*p* < 0.05)

*CEA* carcinoembryonic antigen, *CRM* circumferential resection margin, *EMVI* extramural venous invasion, *LNM* lymph node metastasis, *LVI* lymph vascular invasion, *PI* perineural invasion, *PR* peritoneal reflection, *TDs* tumour deposits, *IQR* interquartile range, *SD* standard deviation

*The predicted tasks: pT, pLNM, and pDFS

### Radiomics feature selection

**Fig. 3:**
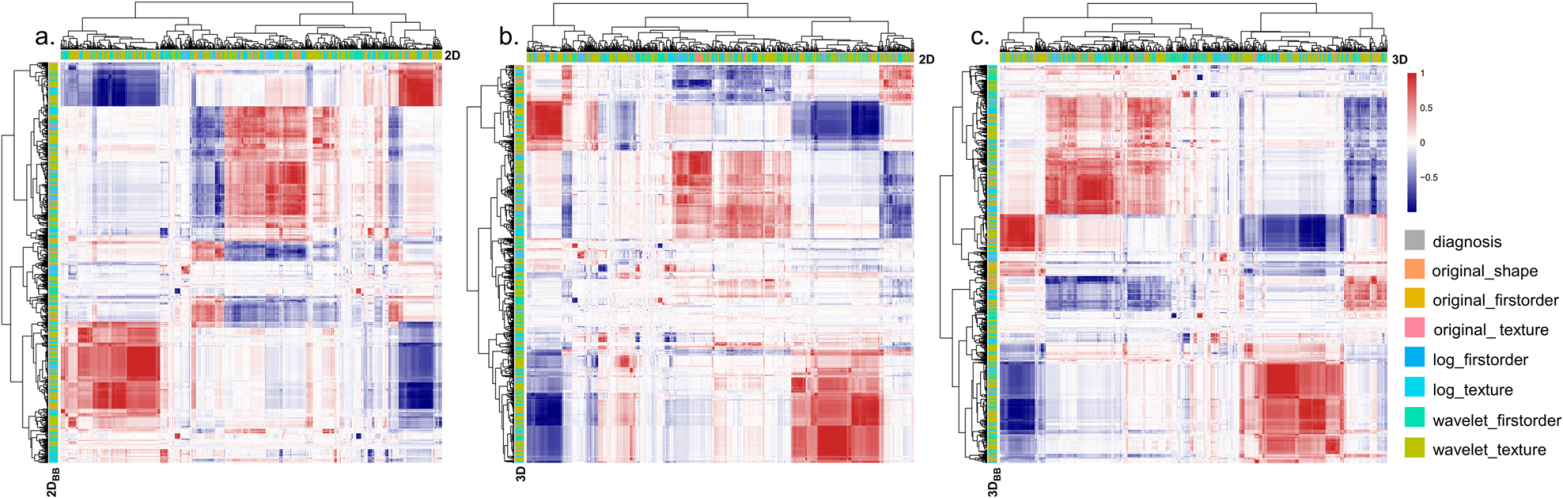
Heatmaps of radiomic features cluster. Redder regions indicate strong positive correlations, and bluer areas indicate strong negative correlations. There were 10.9% between 2D and 3D features (**a**), 10.8% between 2D and 2D_BB_ features (**b**), and 11.3% between 3D and 3D_BB_ features (**c**) subset has a significant correlation (|correlation coefficient| > 0.6)

A total of 1135 features were extracted from each of the four types of ROI. We analysed the features for potential relevance using the k-means unsupervised clustering algorithm, and presented the resulting visual heatmaps in Fig. 3. Based on the ICC test, 93.0% of 2D and 2D_BB_ features and 91.1% of 3D and 3D_BB_ features, were high-robust and included in the subsequent analysis. After univariate factor analysis, Lasso-Logistic and Lasso-Cox, the final feature sets consist of 12, 13, 16, and 9 features (2D^pT^, 3D^pT^, ${2\text{D}}_{\text{BB}}^{\text{pT}}$, ${3\text{D}}_{\text{BB}}^{\text{pT}}$, respectively); 17, 14, 17, and 10 features (2D^pLNM^, 3D^pLNM^, ${2\text{D}}_{\text{BB}}^{\text{pLNM}}$, ${3\text{D}}_{\text{BB}}^{\text{pLNM}}$, respectively); 12, 13, 15, and 13 features (2D^pDFS^, 3D^pDFS^, ${2\text{D}}_{\text{BB}}^{\text{pDFS}}$, ${3\text{D}}_{\text{BB}}^{\text{pDFS}}$, respectively). The results of feature selection and the corresponding coefficients were outlined in Supplementary Result S1.

### Evaluation of radiomics models

**Fig. 4:**
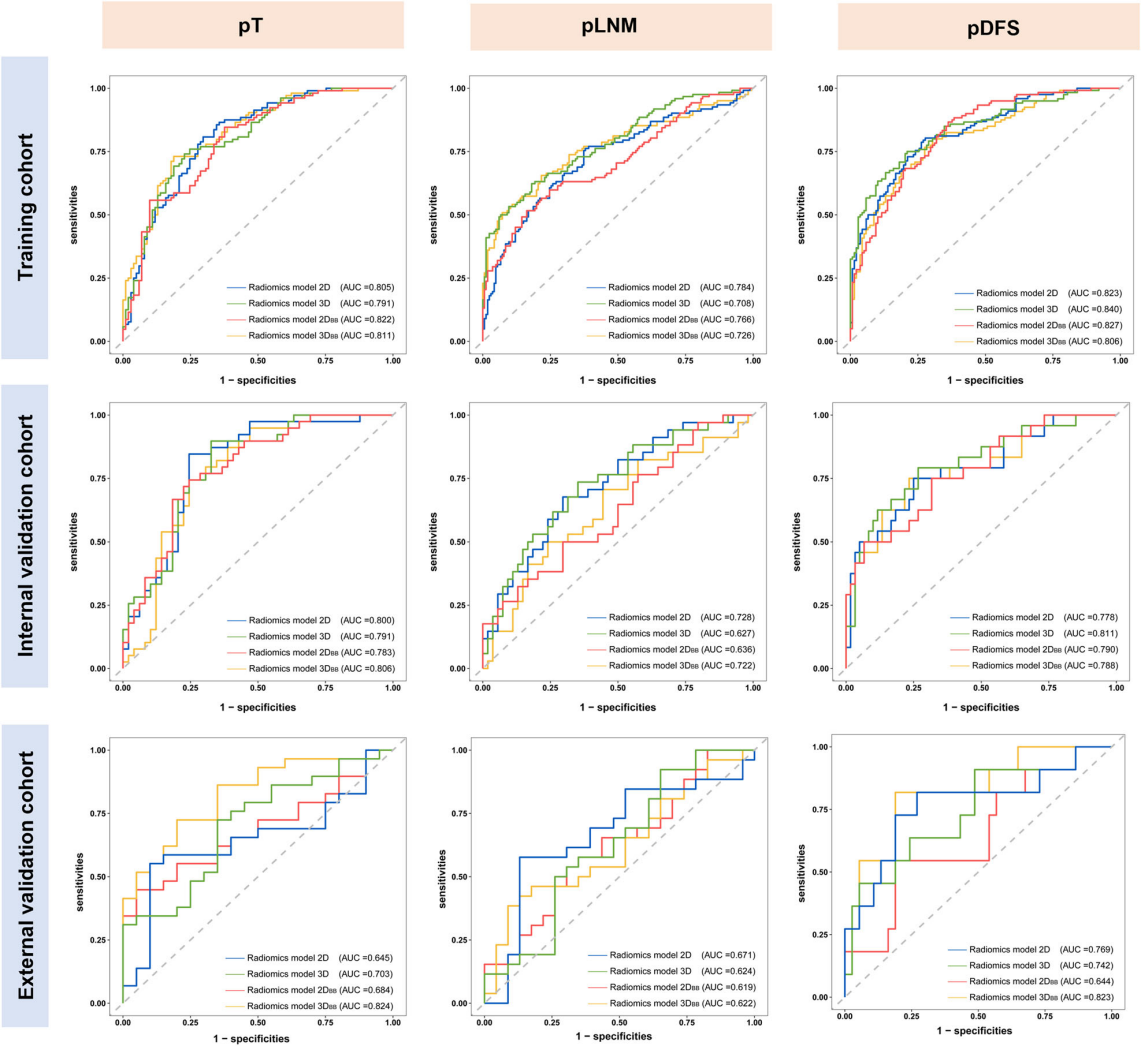
Receiver operating characteristic curves of the radiomics models in the three tasks

Table 2 and Fig. 4 show the results for the training, internal validation and external validation cohorts. In the internal validated based on radiomics models of four annotations, the AUCs ranged from 0.783 (95% CI: 0.683–0.864) to 0.806 (95% CI: 0.708–0.882) for the pT task and 0.627 (95% CI: 0.518–0.728) to 0.728 (95% CI: 0.622–0.817) for the pLNM task; and the AUCs ranged from 0.778 (95% CI: 0.669–0.888) to 0.811 (95% CI: 0.705–0.917). for the pDFS task with the C-indexes ranged from 0.687 (95% CI: 0.679–0.795) to 0.739 (95% CI: 0.691–0.784). In the four annotation comparisons for each predicting task, the 3D^pLNM^ and ${3\text{D}}_{\text{BB}}^{\text{pLNM}}$ showed significant differences only in the internal validation with *p* = 0.0188 (95% CI: 0.0157–0.1740). In the external validation cohorts, the AUCs ranged from 0.645 (95% CI: 0.495–0.776) to 0.824 (95% CI: 0.689–0.918) for the pT task and 0.619 (95% CI: 0.469–0.754) to 0.671 (95% CI: 0.522–0.798) for the pLNM task; and the AUCs ranged from 0.644 (95% CI: 0.455–0.832) to 0.769 (95% CI: 0.582–0.949) for the pDFS task with the C-indexes ranged from 0.646 (95% CI: 0.491–0.801) to 0.764 (95% CI: 0.635–0.893). No significant differences were observed between the four annotations for each task in the external validation.

**Table 2:** Performance of the radiomics models constructed based on different annotation methods

Tasks		2D	3D	2D_BB_	3D_BB_
**pT**	Training cohort AUC	0.805 [0.744–0.857]	0.791 [0.729–0.845]	0.822 [0.763–0.872]	0.811 [0.751–0.862]
	Internal validation cohort AUC	0.800 [0.701–0.877]	0.791 [0.691–0.871]	0.783 [0.683–0.864]	0.806 [0.708–0.882]
	External validation cohort AUC	0.645 [0.495–0.776]	0.703 [0.556–0.825]	0.684 [0.536–0.810]	0.824 [0.689–0.918]
**pLNM**	Training cohort AUC	0.784 [0.731–0.830]	0.708 [0.652–0.760]	0.766 [0.713–0.814]	0.726 [0.671–0.777]
	Internal validation cohort AUC	0.728 [0.622–0.817]	0.627 [0.518–0.728]	0.636 [0.526–0.736]	0.722 [0.617–0.812]
	External validation cohort AUC	0.671 [0.522–0.798]	0.624 [0.474–0.758]	0.619 [0.469–0.754]	0.622 [0.472–0.757]
**pDFS**	Training cohort AUC	0.811 [0.759–0.863]	0.840 [0.791–0.888]	0.827 [0.778–0.876]	0.806 [0.753–0.859]
	Internal validation cohort AUC	0.778 [0.669–0.888]	0.811 [0.705–0.917]	0.791 [0.680–0.902]	0.788 [0.673–0.902]
	External validation cohort AUC	0.769 [0.582–0.949]	0.742 [0.567–0.917]	0.644 [0.455–0.832]	0.742 [0.567–0.917]
	Training cohort C-index	0.726 [0.679–0.773]	0.763 [0.720–0.806]	0.731 [0.688–0.774]	0.714 [0.669–0.760]
	Internal validation cohort C-index	0.687 [0.579–0.795]	0.739 [0.691–0.784]	0.732 [0.627–0.837]	0.726 [0.627–0.825]
	External validation cohort C-index	0.675 [0.508–0.842]	0.703 [0.554–0.852]	0.646 [0.491–0.801]	0.764 [0.635–0.893]

Data represent AUC with 95% CI in parentheses. The predicted tasks: pT, pLNM, and pDFS. 2D: detailed annotation based on maximum tumour area level; 3D: detailed annotation based on whole tumour; 2D_BB_: bounding box annotation based on maximum tumour area level; 3D_BB_: bounding box annotation based on whole tumour

**Fig. 5:**
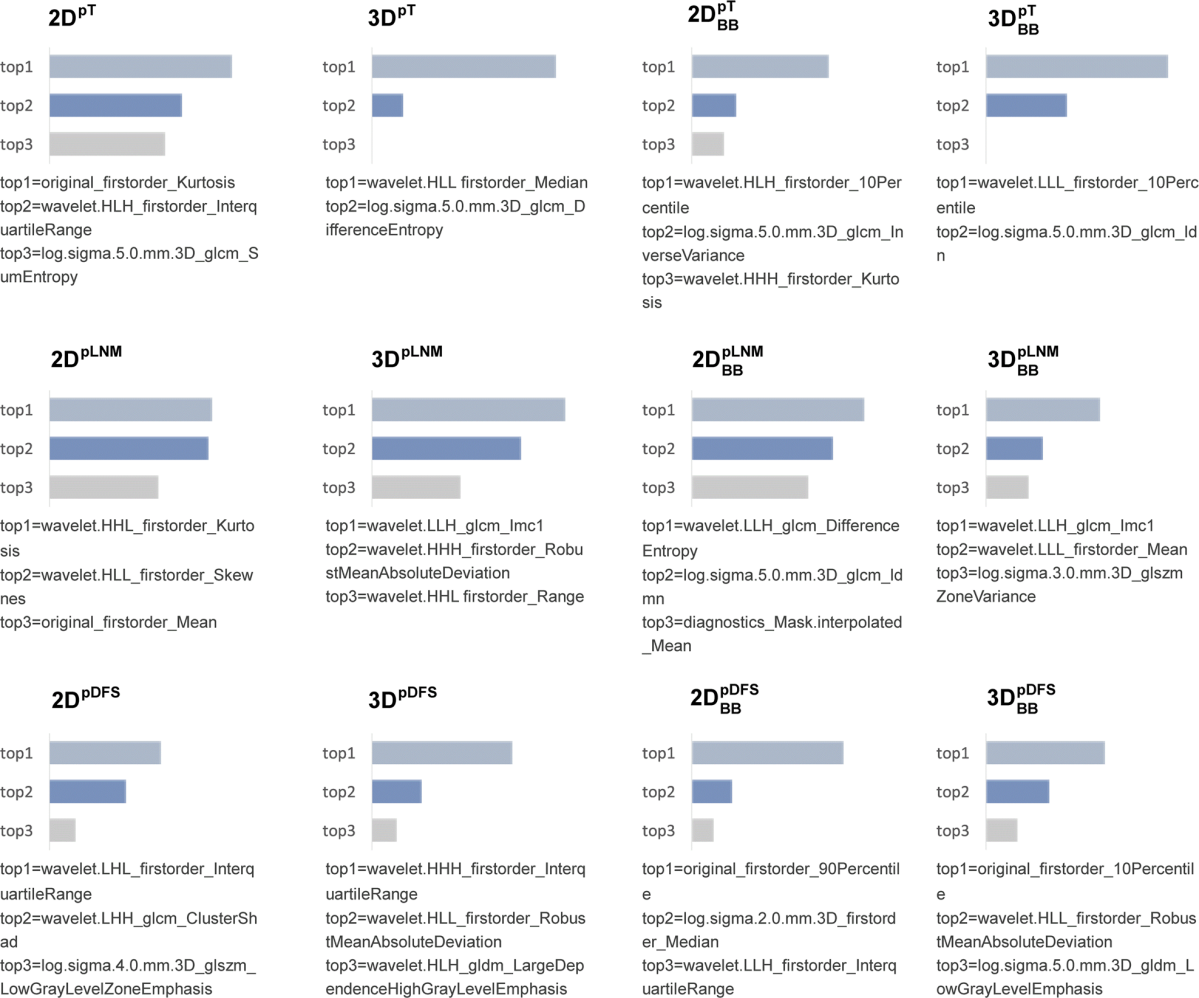
The top three important features in each model. Calculate the top three important features that were significant in the multivariable analysis (In the multivariate analysis of pT tasks, only two features of 3D and 3D_BB_ were significant). The *p*-value was calculated using the Wald test (*p* < 0.005)

We identified the three most important features in each model through feature importance analysis. As shown in Fig. 5, the first-order features are present in most feature sets and play a dominant role in characterising tumour heterogeneity for our study. Firstorder_RobustMeanAbsoluteDeviation, and glcm_DifferenceEntropy, along with some feature types such as LoG filter features, also appear multiple times.

### Development, performance, and internal validation of combined models

Table 3 shows the result of statistically significant clinical factors. In the pT task, we finally incorporated combined models construction in the form of nomograms containing tumour T-stage by MRI (MR-T), tumour length, peritoneal reflex (PR), and Rad-scores (2D^pT^, 3D^pT^, ${2\text{D}}_{\text{BB}}^{\text{pT}}$,$\,{3\text{D}}_{\text{BB}}^{\text{pT}}$, respectively); In the pLNM task, the combined models contained LNM assessed by MRI (MR-LNM), tumour length, PR and Rad-scores (2D^pLNM^, 3D^pLNM^, ${2\text{D}}_{\text{BB}}^{\text{pLNM}}$, ${3\text{D}}_{\text{BB}}^{\text{pLNM}}$, respectively); In the pDFS task, the combined models contained p-T, p-LNM, tumour length, histological grade and Rad-scores (2D^pDFS^, 3D^pDFS^, ${2\text{D}}_{\text{BB}}^{\text{pDFS}}$, and ${3\text{D}}_{\text{BB}}^{\text{pDFS}}$, respectively).

**Table 3:** Univariable and multivariable analyses for each potential clinical characteristic

Tasks	Variables	Univariate analysis		Multivariate analysis	
		OR/HR (95% CI)	*p*-value	OR/HR (95% CI)	*p*-value
**pT**	Age	1.912 (1.098–3.331)	0.022		
	CEA	1.067 (1.017–1.120)	0.009		
	Tumour length	1.047 (1.023–1.072)	< 0.001	1.036 (1.011–1.062)	0.005
	PR	0.564 (0.367–0.867)	0.009	0.578 (0.367–0.915)	0.019
	EMVI	2.768 (1.158–6.616)	0.022		
	MR-T	3.218 (1.800–5.754)	< 0.001	2.440 (1.294–4.637)	0.006
	MR-LNM	2.373 (1.245–4.519)	0.009		
**pLNM**	Tumour length	1.030 (1.010–1.051)	0.003	1.022 (1.001–1.043)	0.040
	PR	0.614 (0.402–0.940)	0.025	0.581 (0.369–0.914)	0.019
	MR-T	2.458 (1.359–4.446)	0.003		
	MR-LNM	2.726 (1.352–5.494)	0.005	2.525 (1.186–5.378)	0.016
**pDFS**	Tumour length	1.034 (1.020–1.047)	< 0.001	1.022 (1.007–1.038)	0.004
	EMVI	2.072 (1.121–3.832)	0.020		
	MR-T	2.219 (1.252–3.933)	0.006		
	p-T	3.476 (1.936–6.240)	< 0.001	2.242 (1.226–4.100)	0.009
	p-LNM	2.895 (1.719–4.876)	< 0.001	2.012 (1.172–3.455)	0.011
	Histologic grade	2.267 (1.343–3.827)	0.002	1.833 (1.084–3.100)	0.024
	TDs	2.132 (1.153–3.943)	0.015		
	LVI	1.833 (1.078–3.117)	0.025		

Parentheses indicate the 95% confidence interval. The predicted tasks: pT, pLNM, and pDFS

*CEA* carcinoembryonic antigen, *EMVI* extramural venous invasion, *LVI* lymph vascular invasion, *PR* peritoneal reflection, *TDs* tumour deposits, *OR* odds ratio, *HR* hazard ratio

**Fig. 6:**
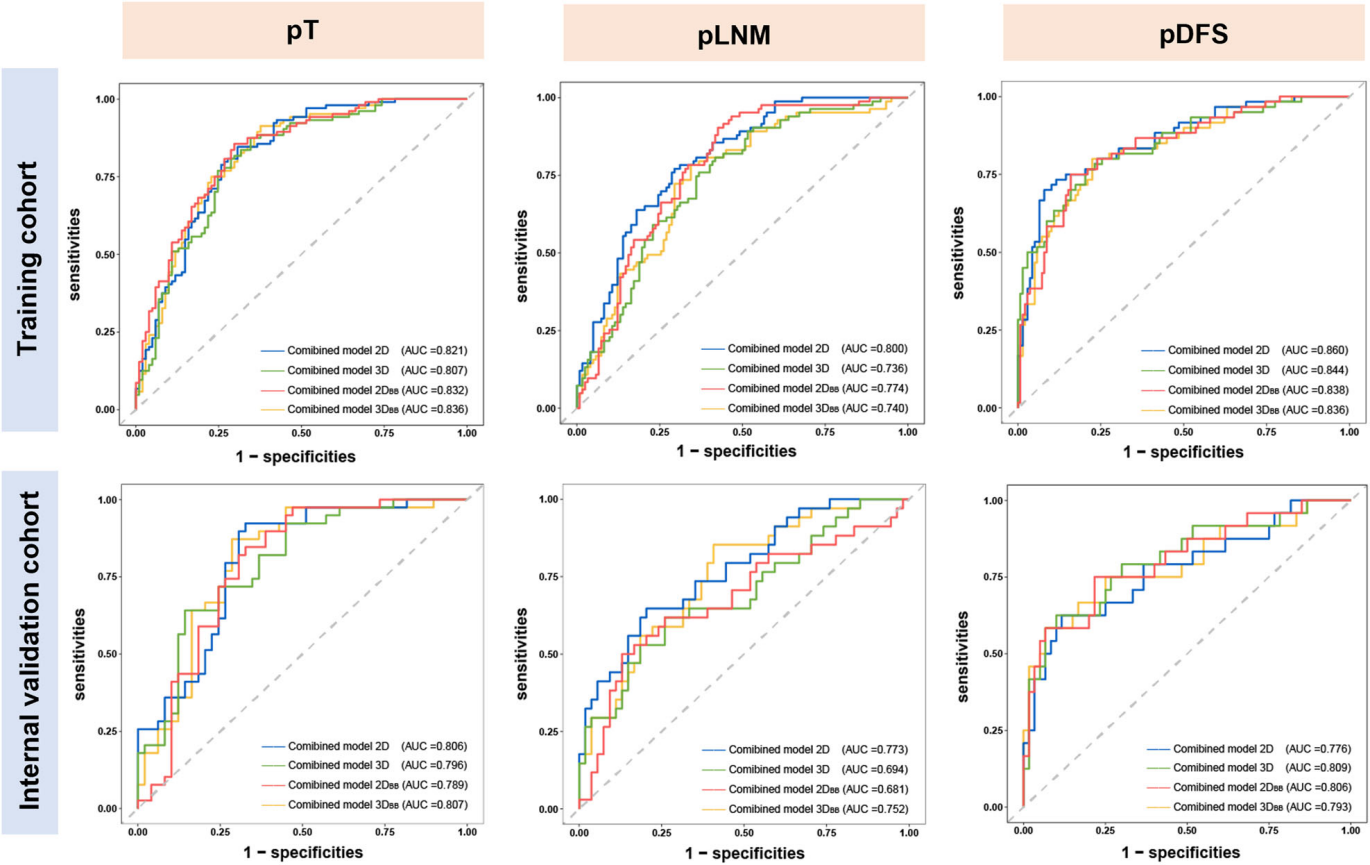
Receiver operating characteristic curves of the combined models in the three tasks

Table 4 and Fig. 6 summarise the predictive performance of the combined models. The comparison of ROCs showed no statistically significant differences between the four annotations in the pT and pLNM tasks. In pDFS tasks, significant differences were found between the 2D^pDFS^ and ${2\text{D}}_{\text{BB}}^{\text{pDFS}}$ models with *p* = 0.0372 (95% CI: 0.0030–0.0998), and between the 2D^pDFS^ and 3D^pDFS^ models with *p* = 0.0380 (95% CI: 0.0029–0.1010).

**Table 4:** Performance of the combined models constructed based on different annotation methods

Tasks		2D	3D	2D_BB_	3D_BB_
**pT**	Training cohort AUC	0.821 [0.761–0.871]	0.807 [0.746–0.858]	0.832 [0.774–0.880]	0.822 [0.762–0.872]
	Internal validation cohort AUC	0.806 [0.708–0.882]	0.796 [0.697–0.874]	0.789 [0.689–0.869]	0.807 [0.709–0.883]
**pLNM**	Training cohort AUC	0.800 [0.739–0.852]	0.736 [0.670–0.795]	0.774 [0.711–0.829]	0.740 [0.674–0.799]
	Internal validation cohort AUC	0.773 [0.671–0.855]	0.694 [0.587–0.788]	0.681 [0.573–0.776]	0.752 [0.649–0.838]
**pDFS**	Training cohort AUC	0.860 [0.802–0.918]	0.844 [0.783–0.906]	0.838 [0.777–0.899]	0.836 [0.774–0.897]
	Internal validation cohort AUC	0.776 [0.658–0.895]	0.809 [0.700–0.919]	0.806 [0.697–0.915]	0.793 [0.675–0.911]
	Training cohort C-index	0.810 [0.757–0.863]	0.805 [0.750–0.860]	0.792 [0.740–0.844]	0.792 [0.738–0.846]
	Internal validation cohort C-index	0.715 [0.611–0.819]	0.749 [0.662–0.836]	0.752 [0.663–0.841]	0.742 [0.647–0.837]

Data represent AUC with 95% CI in parentheses. The predicted tasks: pT, pLNM, and pDFS. 2D: detailed annotation based on maximum tumour area level; 3D: detailed annotation based on whole tumour; 2D_BB_: bounding box annotation based on maximum tumour area level; 3D_BB_: bounding box annotation based on whole tumour

The cumulative event curves of recurrence in patients were stratified according to 2D^pDFS^, 3D^pDFS^, ${2\text{D}}_{\text{BB}}^{\text{pDFS}}$, and ${3\text{D}}_{\text{BB}}^{\text{pDFS}}$ categories, as shown in Supplementary Fig. S.2. The analysis revealed significant associations between all models and recurrent risk (*p* = 0.016, *p* < 0.001, *p* < 0.001, *p* < 0.001, respectively). The calibration curves confirm the excellent calibration of all combined models (Supplementary Fig. S.3). The DCAs indicate that all combined models generate a higher net benefit within a certain range compared to the all/no-intervention strategy (Supplementary Fig. S.4). This demonstrates the favourable clinical utility of the combined models in aiding clinicians to determine the T-stage, LNM, and DFS in rectal cancer patients.

## Discussion

T-stage, LNM, and prognosis of rectal cancer are perennial concerns for patients, and T2WI offers the most valuable diagnostic method for evaluating these critical indicators [24]. Radiomics plays an important role in preoperative staging and postoperative prognostic assessment of rectal cancer, but there has been no standard answer for the choice of tumour annotation methods [25]. Therefore, in this study, four T2WI-based radiomics annotations were used to predict rectal cancer pT, pLNM, and pDFS. The results showed that the radiomics models based on 2D and bounding box annotation had comparable predictive performance in unbalanced internal and external validation cohorts compared to 3D and detailed annotation along tumour boundaries and demonstrated the high robustness of radiomics models. The predictive performance was further improved by combining clinical risk factors and again showed comparable predictive performance of the four annotations in the internal validation cohort.

Radiomics has been extensively applied in medical images, especially in oncology imaging. The establishment of the METRICS scores, the CLEAR, and checklists have provided a standard set of criteria to assess the methodological rigour of radiomics studies, thereby improving radiomics clarity and reproducibility [26, 27]. With the increasing refinement of radiomics standards, the changes brought about by tumour annotation to radiomics have gradually gained attention [28]. While recent studies generally lean towards 3D annotation for its comprehensive depiction of tumours [29, 30], some research comparing 2D and 3D annotations has found 2D annotation to offer comparable stability and repeatability [16, 31–33]. Our results showed similar predictive performance and higher repeatability with 2D annotation. This makes it potentially ideal for rectal cancer studies due to its balance of prediction accuracy, labour, and time efficiency.

Our study found that 2D_BB_ and 3D_BB_ annotations did not show significant prediction performance compared to detailed annotations along tumour boundaries. The detailed annotation takes 30 to 60 s, while bounding box requires less than 10 s [17]. Time-reducing is essential for dealing with managing large datasets and complex scenarios like deep learning [6, 34]. Some studies have also reported that using bounding box annotation can somewhat mitigate the FOV scaling effect [35]. Therefore, bounding box could be more suitable for widespread application. It is noteworthy that the peri-tumour region is also important and is subtly incorporated within bounding box [15, 16, 36]. However, our study found that bounding box annotation did not significantly improve the model’s performance compared to detailed annotations based on tumour boundaries. This may be due to the loss of shape information at the tumour edge. Given the comparable performance and feasibility of bounding box annotation in characterisation and prognostic prediction, it could be considered a viable choice for annotation.

Feature importance analysis showed that certain features consistently emerged as top contributors, regardless of the annotation used. This implies that essential features relevant to specific tasks can be effectively represented by various annotations. Therefore, using simpler annotations can strengthen efficiency without losing vital result-related information. For the pT tasks, we found that features obtained through LoG preprocessing filters consistently ranked as the top three important features in the final feature sets of all four annotations. The LoG preprocessing filter, a derivative filter, is used to emphasise rapidly changing areas in images, specifically edges. These features primarily reflect the edge information of tumours [37]. Based on our further investigations, we looked up T-stage prediction research and also found a similar phenomenon. Hou et al [5] modelled 13 radiomics features on HR-T2WI, including six LoG-filtered features; You and Yin [38] had three LoG-filtered features out of the five selected features. Moreover, the LoG features have been shown in other studies to possess strong stability and reproducibility (ICC = 0.91), which has also been confirmed in our study (ICC2D = 0.92, ICC3D = 0.99) [20]. These results provide strong evidence that LoG features can, to some extent, reflect the depth of infiltration of rectal tumours.

Our study has some limitations. First, the retrospective design and external validation of only 49 cases may introduce bias. We should consider a prospective design and inclusion of data from more centres to validate the results in future studies. Second, only T2WI was analysed in this study, and MRI-derived parameters may have value for tumour heterogeneity. In particular, diffusion-weighted imaging and apparent diffusion coefficient were found to have excellent reproducibility in rectal cancer [39]. Further studies should explore the differences between annotation methods combining multiple sequential images. Despite these limitations, this study still provides some reference value for radiomics annotations.

## Conclusion

This study compared the performance of four different radiomic annotation methods (2D, 3D, 2D_BB_, 3D_BB_) in predicting rectal cancer clinical issues. The results showed that there were no significant differences between these four annotations within radiomics and combined models. Considering the comparability of performance, the use of 2D maximum tumour area and bounding box annotations may be a higher priority than 3D whole tumour annotation and detailed annotation along tumour boundaries.

### Supplementary information

ELECTRONIC SUPPLEMENTARY MATERIAL

## Data Availability

Data are available on reasonable request.

## Abbreviations

AUC: Area under the curve
C-index: Concordance index
DCA: Decision curve analysis
ICC: Interclass correlation coefficient
LoG: Laplacian of Gaussian
MRI: Magnetic resonance imaging
pDFS: Prediction of disease-free survival
pLNM: Prediction of lymph node metastasis
pT: Prediction of tumour T-stage
ROC: Receiver operating characteristic
ROI: Regions of interest
T2WI: T2-weighted imaging
