# Supplementary material for: Different radiomics annotation methods comparison in rectal cancer characterisation and prognosis prediction: a two-centre study
Source: Insights Imaging. 2024 Aug 26;15:211. doi: 10.1186/s13244-024-01795-5 (PMC11347551; doi:10.1186/s13244-024-01795-5)
Supplement: Supplementary file 1 — ELECTRONIC SUPPLEMENTARY MATERIAL [file 13244_2024_1795_MOESM1_ESM.pdf]

Different radiomics annotation methods comparison in rectal cancer characterization and prognosis prediction: A two-centre study

ELECTRONIC SUPPLEMENTARY MATERIAL

Method S1: Clinical information

The electronic medical record system of the First Affiliated Hospital of Wenzhou Medical University was utilized to collect basic demographic data from August 2016 to May 2020 for eligible patients. The data included age, sex, body mass index (BMI), carcinoembryonic antigen (CEA) level, circumferential resection margin (CRM), extramural venous invasion (EMVI), relationship to anterior peritoneal reflection (PR), tumour length, tumour T-stage (MR-T) and lymph node metastasis assessed by magnetic resonance imaging (MR-LNM). The postoperative pathological examination assesses tumour T-stage (p-T) and lymph node metastasis (p-LNM), as well as lymph vascular invasion (LVI), perineural invasion (PI), tumour deposits (TDs), histologic grade, local or distant metastasis, and disease-free survival (DFS) status. Clinical characteristics associated with T-stage and lymph node metastasis were evaluated using univariate and multivariate Logistic regression analyses. Perform both univariate and multivariate Cox regression analyses to identify significant clinical characteristics for evaluating DFS. These remaining important clinical characteristics were used for subsequent combined models construction.

The electronic medical record system of Guangdong Provincial People's Hospital was utilized to collect basic demographic data from January 2016 to December 2018 for eligible patients. The data included age, sex, CEA level, CRM, EMVI, tumour length, MR-T and MR-LNM. The postoperative pathological examination assesses p-T, p-LNM, LVI, PI, TDs, histologic grade, local or distant metastasis, and DFS status.

All patients underwent radical resection. Patients were followed up every 3 months within the first 2 years, every 6 months within the subsequent 3-5 years, and annually thereafter. DFS was defined as the time from the date of surgery to the first local recurrence, distant metastasis, or the last follow-up date. Local recurrence and distant metastasis were assessed using MRI and CT, or confirmed through biopsy.

Method S2 Description of the LASSO method

Lasso-Logistic regression model was used in this study for the prediction of pT and pLNM. The "glmnet" function was used to fit the logistic regression model, with the "family" parameter set to "binomial" to implement binary logistic regression. The regularization parameter "alpha" was set to 1 to ensure the model used L1 regularization. Cross-validation was then performed using the "cv.glmnet" function to determine the optimal regularization parameter "lambda.min". The non-zero coefficients in the model were extracted, and the variables corresponding to these coefficients were selected as key features of the model. To quantify the predictive power of the model, a custom function, "myFun", was defined. This function takes the feature vector "x" and the model coefficients "actCoef" as inputs and calculates their dot product. The result of the dot product serves as the rad-score value for the binary classification model, which is the probability of predicting the positive class. Using the feature vectors obtained from the training set and their coefficient values, the rad-score values were calculated for each sample in the internal and independent external validation cohorts.

Lasso-Cox was used for the prediction of pDFS in this study. Survival time "futime" and survival state "fustat" association were used as model inputs to fit the cox proportional hazards model using the "glmnet" function with the "family" parameter set to "cox" and the alpha parameter set to 1 for L1 regularization. Cross-validation was performed by "cv.glmnet", and we selected the optimal "lambda.min". According to the results of cross-validation, we extracted the non-zero coefficients in the model, and the variables corresponding to these coefficients formed the feature set of the model. Using the "myFun" function, we scored the features in the training and test sets and computed the dot product of the feature vectors "x" and the model coefficients "actCoef" to obtain the risk score and rad-score. Using the feature vectors obtained from the training set and their coefficient values, the rad-score values were calculated for each sample in the internal and independent external validation cohorts.

The optimal hyperparameter λ values and coefficients for Lasso-Logistic and Lasso-Cox are determined as shown in **Fig. S.1**.

Result S1: The features selected for modelling

We extracted 1135 features from each of the four types of ROIs using the open-source software PyRadiomics. These included: diagnostic features (n=3), first-order statistical features (n=234), shape features (n=14), and texture features (n=884). The specific process of feature screening is as follows:

(1) With ICC>75% as a requirement, 101 cases (8.9%) with low robustness features were removed from the 3D annotation, while 1034 cases (91.1%) with high robustness features were retained. The aforementioned culled feature types will be applied to both the 3D and $3D_{BB}$ feature sets. Consequently, 80 cases (7.0%) of low-robustness features were removed from our 2D annotation, and 1055 cases (93.0%) of features with high robustness were retained. The excluded feature types are applied to both 2D and $2D_{BB}$ feature sets, and the last four retained high-robustness feature sets are analyzed for subsequent analysis.

(2) After performing single-factor analysis, there are 86 remaining features in $2D^{pT}$, 88 remaining features in $3D^{pT}$, 121 remaining features in $2D_{BB}^{pT}$, 94 remaining features in $3D_{BB}^{pT}$, 89 remaining features in $2D^{pLNM}$, 74 remaining features in $3D^{pLNM}$, 51 remaining features in $2D_{BB}^{pLNM}$, 64 remaining features in $3D_{BB}^{pLNM}$, 75 remaining features in $2D^{pDFS}$, 140 remaining features in $3D^{pDFS}$, 75 remaining features in $2D_{BB}^{pDFS}$, and 132 remaining features in $3D_{BB}^{pDFS}$.

(3) After using LASSO, a group of key features for 3 tasks and 4 annotation types of ROI were determined. As shown in **Table S.2**, the final feature set consists of 12 features for $2D^{pT}$; 13 features for $3D^{pT}$, 16 features for $2D_{BB}^{pT}$, 9 features for $3D_{BB}^{pT}$, 17 features for $2D^{pLNM}$, 14 features for $3D^{pLNM}$, 17 features for $2D_{BB}^{pLNM}$, 10 features for $3D_{BB}^{pLNM}$, 12 features for $2D^{pDFS}$, 13 features for $3D^{pDFS}$, 15 features for $2D_{BB}^{pDFS}$, and 13 features for $3D_{BB}^{pDFS}$.

Result S2: IDI analysis of the incremental value of radiomics in pT and pLNM

To verify the incremental value of the radiomics model compared with radiological assessment in pT and pLNM tasks, we used the integrated discrimination improvement index (IDI) to deeply analyze the predictive gain of the radiomics model. Specifically, we established integrated models integrating rad-score and radiological assessment by Logistic regression. The receiver operating characteristic (ROC) curve was used to show the comparison of the diagnostic effect between the integrated models and the radiological assessment, as shown in **Fig. S.5**. Subsequently, we quantified the gain of the radiomics on the predictive ability of the models by the IDI test. As shown in **Table S.3**, it was observed that the IDI values were all greater than 0, indicating that the addition of the rad-score brought positive improvement to the model. The larger the IDI value, the stronger the predictive ability of the new model compared with the baseline model. The results showed that the gains of all radiomics scores constructed based on different annotation methods were statistically significant (p<0.0001). This further confirms the validity and superiority of radiomics models in predicting pathological tumour T-stage and LNM.

Table S.1 The MRI image acquisition parameters of the two centres

Hospital	The First Hospital of Wenzhou Medical University (Centre 1, n=293)			Guangdong Provincial People's Hospital (Centre 2, n=49)		
**Scanner**	Philips 3.0T (Achieva)			Philips 3.0T (Ingenia)		
**Sequence**	**T2WI**	**DWI**	**DCE**	**T2WI**	**DWI**	**DCE**
**Field of view (mm2)/(mm)**	230×152	380×285	300×200	100	74	121
**Matrix**	460×304	128×304	376×252	464×461	108×105	280×278
**Slices**	24	24	100	24	36	80
**Section thickness (mm)**	3	4	4	4	6	4
**Slice gap (mm)**	1	1	-1.5	5.1	7	2
**TE (ms)**	80	shortest	2.4	141.12	65.658	1
**TR (ms)**	3000-5000	3000	shortest	4300	922.18	4

DWI = diffusion-weighted imaging; T2WI = T2-weighted imaging; DCE = dynamic contrast-enhanced sequence. Only T2WI sequences were analyzed in this study.

Table S.2 The feature sets selected for radiomics modelling

Tasks	pT	coef	pLNM	coef	pDFS	coef
2D	diagnostics_Mask.interpolated_Mean	-0.43039	original_firstorder_Mean	-0.43031	diagnostics_Mask.interpolated_Mean	-0.01808
	original_firstorder_Mean	-6.54346e-09	diagnostics_Mask.interpolated_Mean	-2.21817e-08	original_firstorder_Mean	-8.41022e-17
	wavelet.HLH_firstorder_InterquartileRange	0.50929	wavelet.LLL_firstorder_Mean	-4.79910e-05	log.sigma.2.0.mm.3D_firstorder_Skewness	0.13261
	original_firstorder_RootMeanSquared	-0.0472	wavelet.HLH_firstorder_90Percentile	0.00783	log.sigma.4.0.mm.3D_glszm_LowGrayLevelZoneEmphasis	-0.15951
	log.sigma.5.0.mm.3D_glcm_DifferenceEntropy	0.12224	wavelet.HLL_firstorder_Skewness	0.63185	wavelet.LLH_firstorder_90Percentile	0.00115
	wavelet.HLL_firstorder_Mean	0.08196	wavelet.LHH_glcm_JointAverage	-0.286936	wavelet.LLH_firstorder_RobustMeanAbsoluteDeviation	0.10633
	log.sigma.5.0.mm.3D_glcm_SumEntropy	0.06442	wavelet.LLH_glcm_MaximumProbability	0.03848	wavelet.LHL_firstorder_InterquartileRange	0.28200
	log.sigma.5.0.mm.3D_glszm_SmallAreaEmphasis	0.04331	wavelet.LHH_firstorder_Mean	0.38500	wavelet.LHH_firstorder_Skewness	0.02478
	log.sigma.4.0.mm.3D_firstorder_Skewness	-0.01872	log.sigma.4.0.mm.3D_glszm_GrayLevelNonUniformityNormalized	-0.35625	wavelet.LHH_glcm_ClusterShade	0.35151
	wavelet.HHH_glszm_LowGrayLevelZoneEmphasis	0.11415	wavelet.LHH_glcm_ClusterShade	0.05313	wavelet.HLL_firstorder_InterquartileRange	0.07293
	original_firstorder_Kurtosis	-0.20688	wavelet.LHH_glrlm_LongRunLowGrayLevelEmphasis	0.15649	wavelet.HLL_firstorder_RobustMeanAbsoluteDeviation	0.09742
	wavelet.HLH_firstorder_Median	0.07757	wavelet.HHL_firstorder_Kurtosis	-0.64747	wavelet.LLL_firstorder_Mean	-0.66972
			wavelet.LLH_firstorder_10Percentile	0.35040		
			wavelet.HLL_glcm_Imc2	-0.42231		
			wavelet.LLH_glcm_SumEntropy	-0.15775		
			original_glszm_SmallAreaEmphasis	-0.27819		
			wavelet.LLL_firstorder_RobustMeanAbsoluteDeviation	-0.22760		
3D	wavelet.LHL_firstorder_Median	-0.18339	wavelet.HHH_firstorder_RobustMeanAbsoluteDeviation	0.35854	log.sigma.2.0.mm.3D_glszm_GrayLevelNonUniformityNormalized	-0.0825
	wavelet.HLL_firstorder_Median	-0.34521	wavelet.HLL_firstorder_Median	-0.05722	log.sigma.4.0.mm.3D_firstorder_Maximum	0.90306
	wavelet.HHH_firstorder_90Percentile	0.28564	wavelet.LHL_firstorder_Median	0.06520	log.sigma.4.0.mm.3D_glszm_SmallAreaLowGrayLevelEmphasis	0.01025
	original_firstorder_RootMeanSquared	-0.15693	wavelet.LLH_glcm_Imc1	0.48025	wavelet.LLH_firstorder_InterquartileRange	0.04077
	log.sigma.5.0.mm.3D_glcm_DifferenceEntropy	0.33747	wavelet.LHH_firstorder_Mean	0.43828	wavelet.LLH_firstorder_Skewness	0.05994
	log.sigma.5.0.mm.3D_glcm_SumEntropy	0.02879	wavelet.LLH_glcm_DifferenceEntropy	0.04934	wavelet.LHH_firstorder_Mean	0.07252
	log.sigma.3.0.mm.3D_firstorder_Minimum	0.14403	wavelet.LLH_glcm_Correlation	-0.00405	wavelet.HLL_firstorder_90Percentile	0.23220
	wavelet.LLH_firstorder_10Percentile	-0.0988	log.sigma.5.0.mm.3D_glcm_Autocorrelation	0.15718	wavelet.HLL_firstorder_Mean	-0.03324
	log.sigma.3.0.mm.3D_glszm_SmallAreaLowGrayLevelEmphasis	0.03073	log.sigma.5.0.mm.3D_gldm_HighGrayLevelEmphasis	0.02890	wavelet.HLL_firstorder_RobustMeanAbsoluteDeviation	0.18438
	wavelet.LLH_glrlm_LongRunHighGrayLevelEmphasis	-0.00941	log.sigma.4.0.mm.3D_glszm_GrayLevelNonUniformityNormalized	-0.26325	wavelet.HLL_glrlm_ShortRunLowGrayLevelEmphasis	-0.15187
	log.sigma.3.0.mm.3D_firstorder_Uniformity	0.09753	original_glszm_SmallAreaHighGrayLevelEmphasis	-0.76042	wavelet.HLH_gldm_LargeDependenceHighGrayLevelEmphasis	0.13629
	log.sigma.3.0.mm.3D_glszm_SmallAreaEmphasis	0.02562	wavelet.HHL_firstorder_Range	-0.25036	wavelet.HHL_glszm_GrayLevelNonUniformityNormalized	0.19781
	wavelet.HHL_glszm_LargeAreaHighGrayLevelEmphasis	-0.14174	log.sigma.3.0.mm.3D_glszm_SmallAreaLowGrayLevelEmphasis	-0.06306	wavelet.HHH_firstorder_InterquartileRange	0.36616
			wavelet.HHL_firstorder_Variance	-0.74897		
$2D_{BB}$	wavelet.LHL_firstorder_InterquartileRange	0.06616	wavelet.LHL_firstorder_Median	-0.37615	original_firstorder_10Percentile	-0.12462
	wavelet.HLH_firstorder_10Percentile	0.12512	wavelet.HLL_firstorder_Median	0.44328	original_firstorder_90Percentile	-1.82651e-08
	wavelet.HLL_firstorder_InterquartileRange	-0.5000	wavelet.HLL_firstorder_90Percentile	0.12052	log.sigma.2.0.mm.3D_firstorder_Median	0.44029
	wavelet.LLL_firstorder_90Percentile	0.40235	diagnostics_Mask.interpolated_Mean	-0.16116	log.sigma.3.0.mm.3D_glrlm_ShortRunLowGrayLevelEmphasis	-0.15453
	original_firstorder_90Percentile	-0.11290	original_firstorder_Mean	-0.00067	log.sigma.3.0.mm.3D_glszm_SmallAreaEmphasis	-9.42889e-05
	wavelet.HLL_firstorder_Median	-3.83918e-09	log.sigma.5.0.mm.3D_glcm_Correlation	-0.68091	log.sigma.5.0.mm.3D_glcm_DifferenceEntropy	0.00784
	log.sigma.5.0.mm.3D_glcm_DifferenceEntropy	-0.01920	log.sigma.4.0.mm.3D_glszm_GrayLevelNonUniformityNormalized	-0.53178	wavelet.LLH_firstorder_InterquartileRange	0.05037
	log.sigma.5.0.mm.3D_glcm_InverseVariance	0.08372	wavelet.LLH_firstorder_Median	-0.43644	wavelet.LLH_firstorder_RobustMeanAbsoluteDeviation	0.04151
	wavelet.LHL_glcm_DifferenceAverage	0.20355	log.sigma.5.0.mm.3D_glcm_Idmn	-0.26829	wavelet.LHL_firstorder_Mean	-0.04081
	log.sigma.5.0.mm.3D_glcm_Idmn	0.11530	log.sigma.3.0.mm.3D_glszm_SmallAreaEmphasis	-0.49468	wavelet.HLL_firstorder_InterquartileRange	0.29989
	wavelet.HHH_glcm_Autocorrelation	0.09201	wavelet.HHH_glszm_HighGrayLevelZoneEmphasis	-0.12968	wavelet.HLL_firstorder_Median	-0.16824
	wavelet.HHH_firstorder_Kurtosis	-0.15083	wavelet.HHH_glszm_LowGrayLevelZoneEmphasis	2.36061e-06	wavelet.HLL_firstorder_RobustMeanAbsoluteDeviation	0.04511
	log.sigma.3.0.mm.3D_firstorder_Uniformity	0.027892	wavelet.LHH_firstorder_Maximum	-0.32157	wavelet.HLH_firstorder_10Percentile	-8.85017e-05
	wavelet.HLH_firstorder_Range	-0.01506	wavelet.LLH_firstorder_10Percentile	0.32338	wavelet.HHH_glszm_HighGrayLevelZoneEmphasis	-0.010135
	wavelet.HLL_glszm_LargeAreaHighGrayLevelEmphasis	-0.01488	wavelet.LLH_glcm_DifferenceEntropy	0.70910	wavelet.HHH_glszm_LowGrayLevelZoneEmphasis	4.12632e-07
	wavelet.LHL_glcm_Idmn	-0.04594	log.sigma.5.0.mm.3D_glcm_DifferenceEntropy	-0.46382		
			wavelet.LHL_gldm_GrayLevelVariance	-0.32270		
$3D_{BB}$	wavelet.LLL_firstorder_10Percentile	-0.65796	wavelet.LHL_firstorder_Median	-0.04256	diagnostics_Mask.interpolated_Mean	-0.04287
	wavelet.LLL_firstorder_Median	-1.60140e-15	wavelet.LLL_firstorder_Mean	-0.32038	original_firstorder_10Percentile	-0.18905
	wavelet.LHH_firstorder_90Percentile	0.26611	wavelet.LLH_glcm_Imc1	0.46231	original_firstorder_90Percentile	-0.60389
	log.sigma.5.0.mm.3D_glcm_InverseVariance	0.21685	log.sigma.5.0.mm.3D_glcm_Correlation	-0.15061	original_glrlm_LowGrayLevelRunEmphasis	-0.00003
	log.sigma.5.0.mm.3D_glcm_Idn	-0.15361	wavelet.HHL_firstorder_RootMeanSquared	-0.23289	original_glrlm_ShortRunLowGrayLevelEmphasis	-0.03859
	wavelet.LLH_firstorder_10Percentile	-0.02573	log.sigma.4.0.mm.3D_glszm_GrayLevelNonUniformityNormalized	-0.08239	original_glszm_LowGrayLevelZoneEmphasis	-0.00039
	wavelet.LHH_firstorder_Median	-0.67307	log.sigma.4.0.mm.3D_glcm_JointAverage	-0.18015	log.sigma.5.0.mm.3D_gldm_LowGrayLevelEmphasis	-0.14508
	wavelet.HHL_firstorder_Maximum	-0.09524	log.sigma.5.0.mm.3D_gldm_LargeDependenceHighGrayLevelEmphasis	-0.00293	wavelet.LLH_firstorder_90Percentile	0.04197
	wavelet.LHH_glszm_LargeAreaHighGrayLevelEmphasis	-0.00836	log.sigma.3.0.mm.3D_glszm_ZoneVariance	-0.26348	wavelet.LLH_glszm_LargeAreaLowGrayLevelEmphasis	-0.02370
			wavelet.HHL_firstorder_Skewness	0.05623	wavelet.LHL_firstorder_Median	-0.19016
					wavelet.HLL_firstorder_RobustMeanAbsoluteDeviation	0.11797
					wavelet.HLL_glszm_SmallAreaLowGrayLevelEmphasis	-0.04101
					wavelet.HHL_firstorder_RootMeanSquared	-0.08589

2D: detailed annotation based on maximum tumour area level; 3D: detailed annotation based on whole tumour. $2D_{BB}$: bounding box annotation based on maximum tumour area level; $3D_{BB}$: bounding box annotation based on whole tumour.

Table S.3 Significance comparison between integrated models and sole radiological assessment

**Models**		**MR-T**		**MR-LNM**	
	AUC [95%CI]	IDI [95%CI]	*p*-value	IDI [95%CI]	*p*-value
Radscore2D^{pT} + MR-T	0.738 [0.686-0.790]	0.0513 [0.0273-0.0752]	**<0.0001**	——	——
Radscore3D^{pT} + MR-T	0.765 [0.718-0.816]	0.0948 [0.0643-0.1254]	**<0.0001**	——	——
Radscore2D$^{pT}_{BB}$ + MR-T	0.788 [0.740-0.837]	0.1252 [0.0915-0.1589]	**<0.0001**	——	——
Radscore3D$^{pT}_{BB}$ + MR-T	0.765 [0.716-0.813]	0.0930 [0.0628-0.1233]	**<0.0001**	——	——
Radscore2D^{pLNM} + MR-LNM	0.759 [0.714-0.807]	——	——	0.1261 [0.0908-0.1613]	**<0.0001**
Radscore3D^{pLNM} + MR-LNM	0.720 [0.668-0.774]	——	——	0.0763 [0.0484-0.1042]	**<0.0001**
Radscore2D$^{pLNM}_{BB}$ + MR-LNM	0.718 [0.666-0.773]	——	——	0.0592 [0.0346-0.0839]	**<0.0001**
Radscore3D$^{pLNM}_{BB}$ + MR-LNM	0.726 [0.673-0.782]	——	——	0.0551 [0.0332-0.0770]	**<0.0001**

Boldface indicates statistical significance (p<0.05).

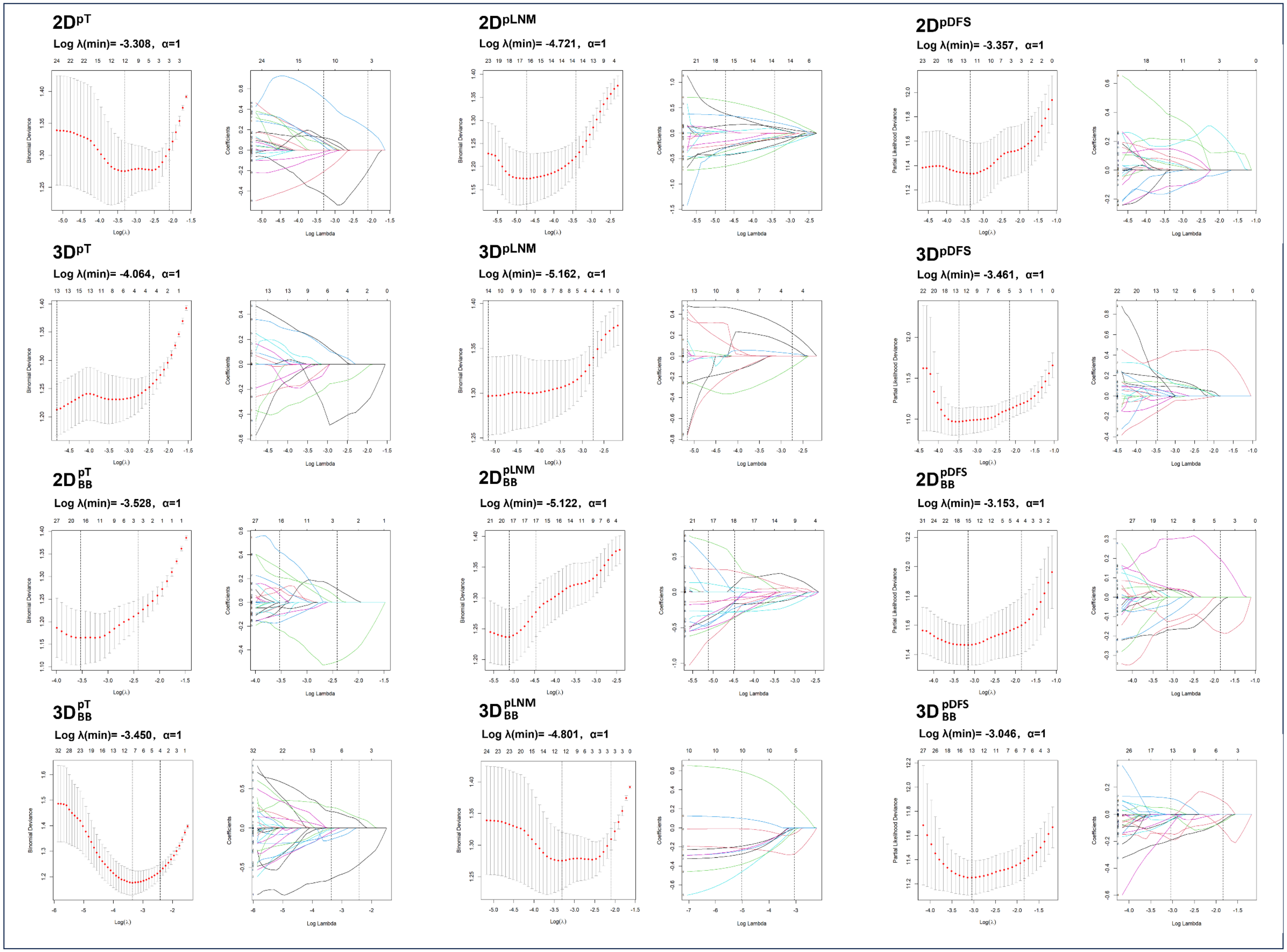

Fig. S.1 The optimal hyperparameter λ values were identified through 10-fold cross-validation during the modelling of 12 radiomics features. The lowest values identified were determined to be the feature that best matched the true results. Lasso-Logistic was employed for the pT and pLNM prediction tasks, while Lasso-Cox was utilized for the pDFS task. The LASSO regression model identified radiomics features with non-zero coefficients.

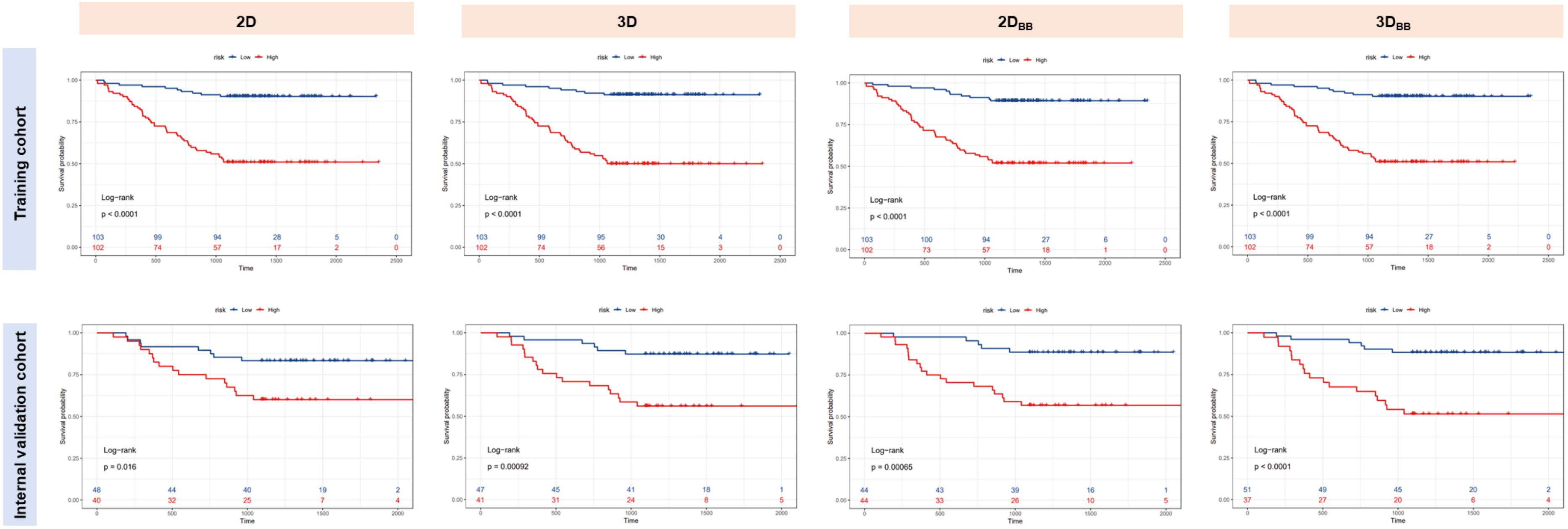

Fig. S.2 Kaplan–Meier curves based on the combined models. The *p*-value was calculated using a two-sided Log-rank test. The predicted tasks: pT, pLNM, and pDFS. 2D: detailed annotation based on maximum tumour area level; 3D: detailed annotation based on whole tumour. $2D_{BB}$: bounding box annotation based on maximum tumour area level; $3D_{BB}$: bounding box annotation based on whole tumour.

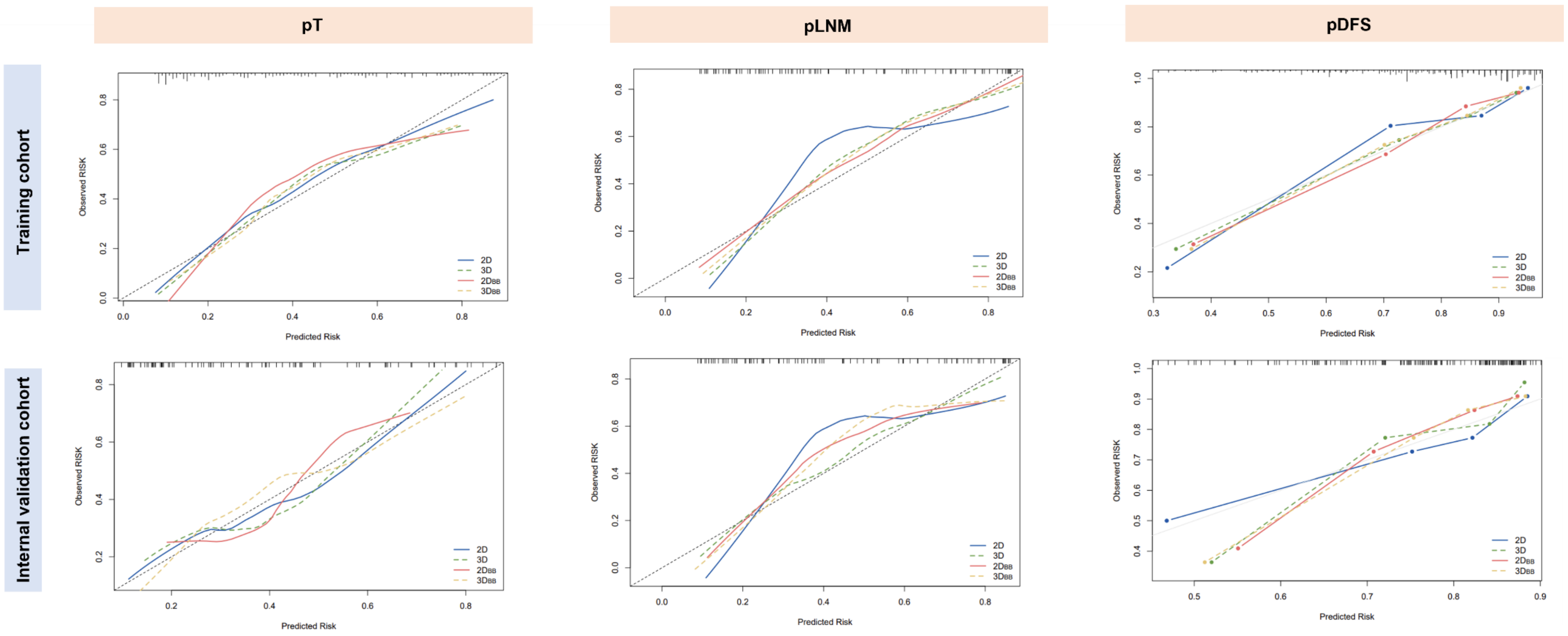

Fig. S.3 Calibration curves of the combined models. In the three tasks, the calibration curves of the combined models all showed good calibration. The predicted tasks: pT, pLNM, and pDFS. 2D: detailed annotation based on maximum tumour area level; 3D: detailed annotation based on whole tumour. $2D_{BB}$: bounding box annotation based on maximum tumour area level; $3D_{BB}$: bounding box annotation based on whole tumour.

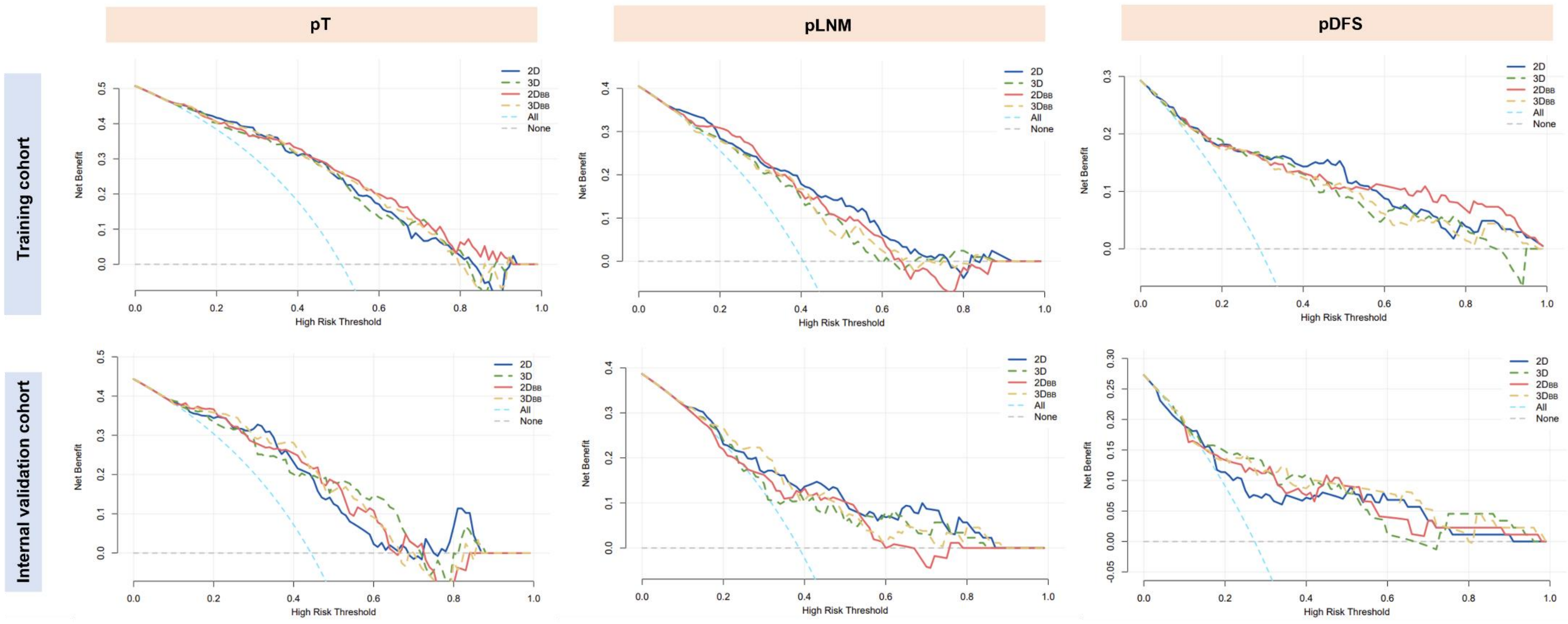

Fig. S.4 Decision curves analysis of the combined models. The x-axis represents the threshold probability, whereas the y-axis illustrates the net benefit. The decision curves indicate that all combined models generate a higher net benefit within a certain range compared to the all/no-intervention strategy.
The predicted tasks: pT, pLNM, and pDFS. 2D: detailed annotation based on maximum tumour area level; 3D: detailed annotation based on whole tumour. $2D_{BB}$: bounding box annotation based on maximum tumour area level; $3D_{BB}$: bounding box annotation based on whole tumour.

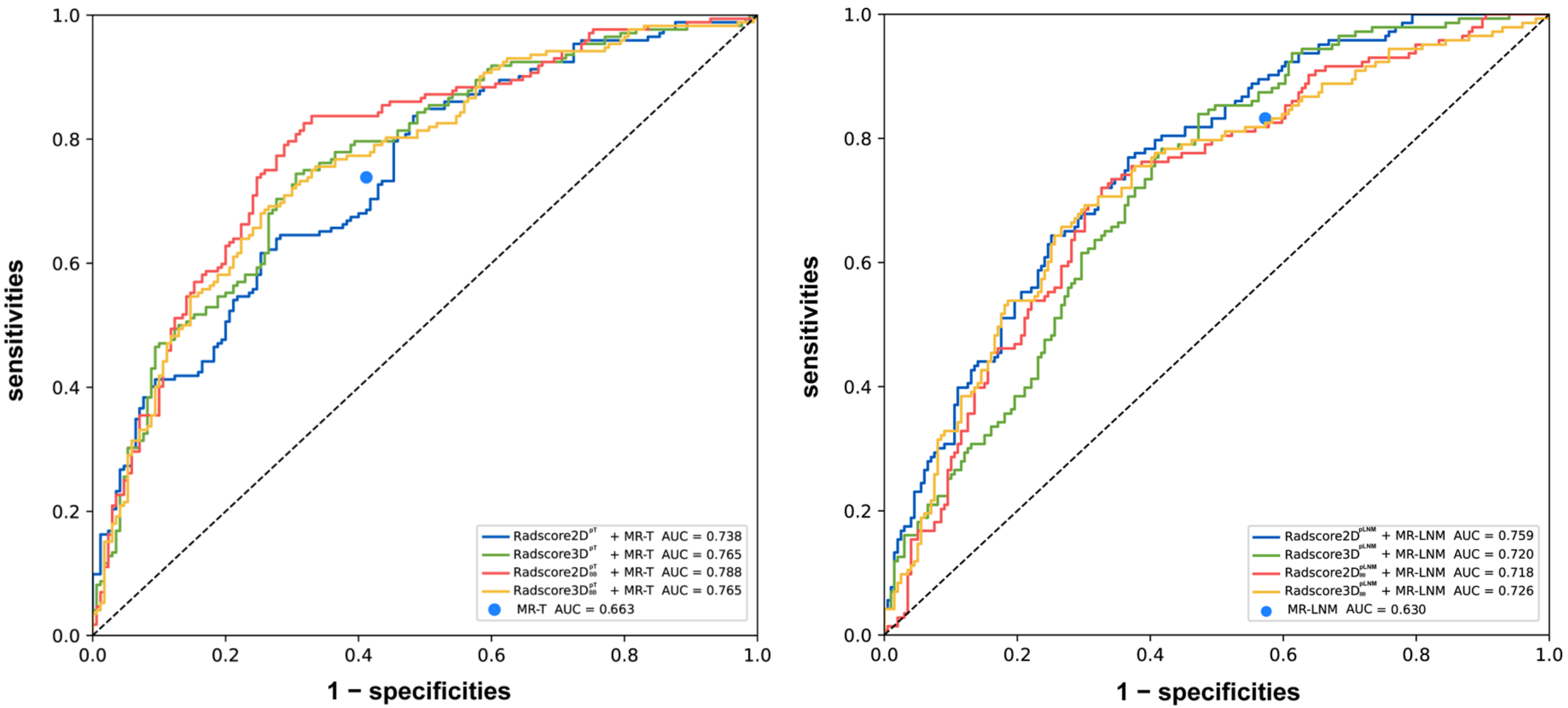

Fig. S.5 Receiver operating characteristic curves for integrated modelling and radiological assessment in the pT and pLNM tasks.
